# Diacylglycerol Kinase β Knockout Mice Exhibit Lithium-Sensitive Behavioral Abnormalities

**DOI:** 10.1371/journal.pone.0013447

**Published:** 2010-10-18

**Authors:** Kenichi Kakefuda, Atsushi Oyagi, Mitsue Ishisaka, Kazuhiro Tsuruma, Masamitsu Shimazawa, Koichi Yokota, Yasuhito Shirai, Kyoji Horie, Naoaki Saito, Junji Takeda, Hideaki Hara

**Affiliations:** 1 Molecular Pharmacology, Department of Biofunctional Evaluation, Gifu Pharmaceutical University, Gifu, Japan; 2 Carna Biosciences, In., Kobe, Japan; 3 Biosignal Research Center, Kobe University, Kobe, Japan; 4 Department of Social and Environmental Medicine, Graduate School of Medicine, Osaka University, Suita, Japan; Pennsylvania State University, United States of America

## Abstract

**Background:**

Diacylglycerol kinase (DGK) is an enzyme that phosphorylates diacylglycerol (DG) to produce phosphatidic acid (PA). DGKβ is widely distributed in the central nervous system, such as the olfactory bulb, cerebral cortex, striatum, and hippocampus. Recent studies reported that the splice variant at the COOH-terminal of DGKβ was related to bipolar disorder, but its detailed mechanism is still unknown.

**Methodology/Principal Findings:**

In the present study, we performed behavioral tests using DGKβ knockout (KO) mice to investigate the effects of DGKβ deficits on psychomotor behavior. DGKβ KO mice exhibited some behavioral abnormalities, such as hyperactivity, reduced anxiety, and reduced depression. Additionally, hyperactivity and reduced anxiety were attenuated by the administration of the mood stabilizer, lithium, but not haloperidol, diazepam, or imipramine. Moreover, DGKβ KO mice showed impairment in Akt-glycogen synthesis kinase (GSK) 3β signaling and cortical spine formation.

**Conclusions/Significance:**

These findings suggest that DGKβ KO mice exhibit lithium-sensitive behavioral abnormalities that are, at least in part, due to the impairment of Akt-GSK3β signaling and cortical spine formation.

## Introduction

By activation of Gq-coupled receptors, phospholipase C (PLC) is activated and hydrolyzes phosphoinositol 4,5-bisphosphate (PIP_2_) into diacylglycerol and inositol 1,4,5-trisphosphate (IP_3_). Diacylglycerol (DG) regulates the functions of several enzymes, such as protein kinase C (PKC), Ras guanyl nucleotide-releasing protein, chimerins, and transient receptor potential cation channel C 3/6 [1], [2], [3], [4]. Subsequently, DG is phosphorylated by diacylglycerol kinase (DGK) to produce phosphatidic acid (PA), an important lipid second messenger which regulates several key enzymes, including the target of rapamycin, atypical PKC and phosphatidylinositol-4-phosphate 5-kinase (PI4P5-kinase) [1], [2], [3], [4]. Therefore, DGK plays an important role in regulating many kinds of intracellular signaling.

DGKβ, a member of the DGK family, is widely distributed in the brain, particularly in the olfactory bulb, cerebral cortex, striatum, and hippocampus [5]. DGKβ is mainly expressed in neurons, and its expression rapidly increases after 14 days of age in the rat, which is coincident with synapse formation in the brain [6]. In addition, we and other researchers previously demonstrated that DGKβ regulates spine formation by regulating lipids, and DGKβ knockout (KO) mice show impaired memory [7], [8]. Furthermore, it was reported that the splice variant at the COOH-terminal of DGKβ was related to bipolar disorder [9]. These results indicate that DGKβ plays an essential role in neurons, and its functions are closely related to neurodegenerative disease and mental diseases.

Bipolar disorder is mental disorder characterized by unusual shifts in mood from the heights of mania to the depths of depression. The lifetime prevalence of this disease is approximately 1%, and epidemiological surveys suggested that some genes such as Ankyrin 3 and DGKη contribute to the onset of this disorder [10], [11]. A common treatment for bipolar disorder is lithium. This drug is comparatively effective, but it has a high incidence of adverse effects, in particular because lithium has a narrow therapeutic window. Therefore, new drugs which have both efficacy and fewer side effects are needed. The purpose of our present work was to investigate the involvement of DGKβ in bipolar disorder. For this purpose, we have developed DGKβ KO mice [8] and performed behavioral, pharmacological and morphological tests using these mice.

## Materials and Methods

### Animals

DGKβ KO (C57BL6/N) mice were generated using the *Sleeping Beauty* transposon system as described previously [8], [12], [13]. This study was approved by the Animal Experiment Committee of Gifu Pharmaceutical University (permission number; 2008-114, 2009-038, 2009-062, and 2009-331), the Institutional Animal Care and Use committee of Kobe University (permission number; 19-5-02) and the committee for Safe Handling of Living Modified organisms in Kobe University (H19-2). All procedures relating to animal care and treatment conformed to animal care guidelines of these committees. All efforts were made to minimize both suffering and the number of animals used. The animals (male, 10–24 weeks old) were housed at 24±2°C under a 12 hr light-dark cycle (lights on from 8:00 to 20:00) and had ad libitum access to food and water. In all experiments, we used wild-type (WT) littermates as a control group for DGKβ KO mice. Behavioral experiments were performed between 10:00 a.m. and 6:00 p.m. except for the 24 hr home cage locomotor activity test.

### Drug treatments

For an acute treatment, lithium chloride (LiCl, Wako, Osaka, Japan) (100 and 200 mg/kg) and imipramine hydrochloride (Wako) (20 mg/kg) were dissolved in saline; diazepam (Wako) (1 or 3 mg/kg) was suspended in the 0.5% carboxymethylcellulose (Wako) solution. Each drug was injected intraperitoneally (i.p.) 30 min before the test. For chronic treatment, LiCl was mixed into the drinking water at 600 mg/L and given for 10 days [14].

### Locomotor activity in the home cage

To measure locomotor activity in a novel environment, a mouse was placed in a transparent plastic cage (length 24.5× width 17.5× height 12.5 cm) with sawdust bedding on the floor. “Home cage” in this draft means the same cage as they are usually housed, i.e. the same size and same color. Animals were placed in the cages at 12:00 p.m. and left there for a 48 hr period. Locomotion was measured every hour for 1 day after 24 hr accumulation using a digital counter with an infrared sensor (NS-ASS01; Neuroscience, Inc., Tokyo, Japan). Movement of the mice was detected by the infrared ray sensor on the basis of released infrared rays associated with their temperature. When objects emitting an infrared ray comes across the sensor, the sensor detected the action as locomotor counts.

### Open field test

To assess the effect of a single-dose drug treatment, LiCl (100 and 200 mg/kg), haloperidol (0.1 mg/kg), diazepam (1.0 mg/kg), or imipramine (20 mg/kg) was dissolved in saline or carboxymethyl cellulose, and injected intraperitoneally. After drug treatment, each mouse was placed in the periphery of the open field apparatus (length 30× width 30× height 30 cm). Thirty minutes later, the total distance moved in the arena and the time spent in the center area (length 15 × width 15 cm) were recorded for the 1 hr test session using a computer-operated EthoVision XT system (Noldus, Wageningen, The Netherlands). The number of scratching behaviors was manually counted for the first 10 min of each test session in a blind manner by a single observer (M.I.). For assessment of chronic lithium treatment, LiCl was mixed into the drinking water at 600 mg/L and given for 10 days. Subsequently, each mouse was placed in the periphery of the open field apparatus and recorded the total distance moved in the arena and the time spent in the center area for 1 hr using EthoVision XT.

### Elevated plus maze test

Elevated plus maze test apparatus consisted of two open arms (length 30 × width 5 cm) and two closed arms of the same size, along with a semi-transparent wall (height 15 cm) and central platform (length 5 × width 5 cm). These arms and central platform were elevated 50 cm above the floor. Each mouse was administered diazepam (3 mg/kg, suspended in 0.5% carboxymethylcellulose solution) or vehicle i.p. Thirty-minutes after the drug administration, each mouse was placed in the central platform, facing one of the open arms. During a 10 min test session, mouse behavior was recorded using EthoVision XT. The number of entries into the open and closed arms, the time spent in the open arms, and the number of times falling from the apparatus were scored. This assessment session was conducted two times (pre or post test) before and after the 10 days of LiCl treatment.

### Forced swim test

Mice were placed in a glass cylinder (diameter 10 cm) filled with 10 cm of water (25±1°C) for a period of 6 min; only for the last 5 min was immobility time measured. Each mouse was administered imipramine hydrochloride (20 mg/kg, dissolved in saline) or vehicle i.p. 30 min before the trial. Mice were judged to be immobile when they remained floating passively in the water, making only small movements to keep their heads above the water.

### Tail suspension test

Each mouse was administered imipramine hydrochloride (20 mg/kg, dissolved in saline) or vehicle i.p. Thirty min later, mouse was tail -suspended with an adhesive tape 50 cm above the floor, and their behavior recorded for 7 min. Immobility time was measured for the last 6 min of the test automatically with the aid of the EthoVision XT system. Mice were judged to be immobile when the mobility score of the system was less than 10%.

### Western blot analysis

At 30 min after LiCl (200 mg/kg, i.p.) or haloperidol (0.1 mg/kg, i.p.) administration, each mouse was decapitated, its brain quickly removed from the skull, briefly washed in ice-cold saline, and laid on a cooled (4°C) metal plate where the cortex was rapidly dissected. Brain samples were homogenized in 10 ml/g tissue ice-cold lysis buffer [50 mM Tris-HCl (pH 8.0) containing 150 mM NaCl, 50 mM EDTA, 1% Triton X-100, and protease/phosphatase inhibitor mixture] and centrifuged at 14,000 × g for 40 min at 4°C. An aliquot of 5 µg of protein was subjected to 10% sodium dodecyl sulfate-polyacrylamide gel electrophoresis, with the separated protein being transferred onto a polyvinylidene difluoride membrane (Immobilon-P; Millipore, Billerica, MA, USA). For immunoblotting, the following primary antibodies were used: Anti-phospho-Akt (Ser473) (193H12) rabbit mAb, phospho-Akt (Thr308) (C31E5E) rabbit mAb, Akt rabbit Ab, phospho-GSK3β (Ser9) (5B3) rabbit mAb, GSK3β (27C10) rabbit mAb, phospho-GSK3α/β rabbit Ab, GSK3α/β rabbit Ab (1∶1000 dilution; Cell Signaling, Danvers, MA, USA), monoclonal anti-β-actin (1∶5000 dilution; Sigma Aldrich, St. Louis, MO, USA), and PHF-Tau (AT-8) mAb (1/200 dilution, Thermo scientific, Waltham, MA, USA). The secondary antibody was as follows: HRP-conjugated goat anti-mouse IgG, HRP-conjugated goat anti-rabbit IgG (1∶4000 dilution; Pierce Biotechnology, Rockford, IL, USA). Immunoreactive bands were visualized using Super Signal West Femto Maximum Sensitivity Substrate (Thermo Scientific, Waltham, MA, USA). The band intensity was measured using a Luminescent image analyzer LAS-4000 UV mini (Fujifilm, Tokyo, Japan) and Multi Gauge Ver. 3.0 (Fujifilm). For quantitative analysis, total proteins were used as loading controls for phosphoprotein signal.

### Histological staining

Mice were anesthetized with sodium pentobarbital (50 mg/kg) and perfused with phosphate buffered saline (PBS) (pH 7.4) until the outflow became clear, and was immediately followed by 0.1 M PBS (pH 7.4) containing 4% paraformaldehyde (Wako) for 15 min. Brains were removed and postfixed in the same fixative for 24 hr at 4°C. For cresyl violet staining, brain sections were equilibrated in 25% sucrose solution and quickly frozen in Tissue-Tek O.C.T. (Sakura Finetek, Torrance, CA, USA). Ten mm thick coronal sections (Bregma + 0.5 mm) were stained with cresyl violet (Sigma, St. Louis, MO, USA). For golgi staining, brain sections were immersed in 30% sucrose for 2 to 3 days. The tissue block was placed in 2% potassium dichromate for 2 days at 4°C and then in 2% silver nitrate solution for 2 days at 4°C in the dark. The block was cut into 60 µm thick and placed into distilled water. Finally, the sections were mounted onto slides, dried for 10 min, and dehydrated through 95% alcohol, 100% alcohol, and clear in xylene.

### Statistical analysis

Data are presented as the means ± S.E.M. Statistical comparisons were made by *t*-test or one-way ANOVA followed by Dunnett's test or Tukey's test using Statview version 5.0 (SAS Institute Inc., Cary, NC, USA), with p<0.05 being considered to indicate a statistical significance.

## Results

### DGKβ KO mice exhibited hyperactivity

**Figure 1:**
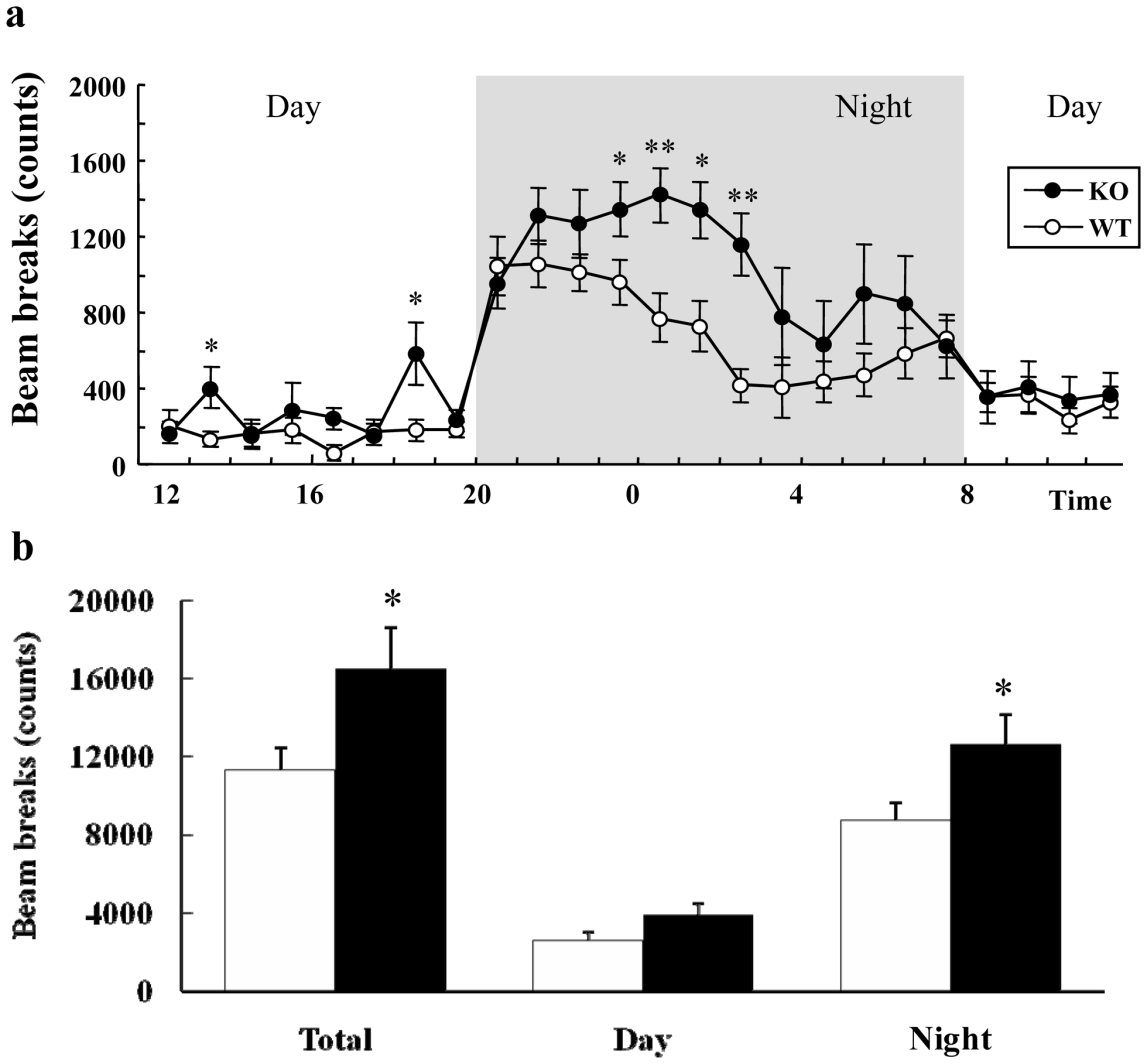
Locomotor activity test of WT and DGKβ KO mice. WT (n = 10) and DGKβ KO (n = 9) mice were placed in individual home cages, and their locomotion was assessed every hour for 24 hr. (a) Locomotor activity throughout the 24-hr period and (b) locomotor activity was analyzed separately during the day and night. ^*^; p<0.05, ^**^; p<0.01 vs. WT mice.

In order to know the effects of DGKβ deficit on the general behavior and circadian rhythm in mice, we first performed locomotor activity tests in the home cage on DGKβ KO mice and their WT littermates. In this test, the activity of DGKβ KO mice was relatively low during the light-phase, and markedly increased just as the dark-phase began, indicating that DGKβ KO mice displayed a normal circadian rhythm (Figure 1a). However, the total (24 hr) and dark-phase locomotor activity counts were larger in KO mice than in WT mice (Figures 1a and b).

**Figure 2:**
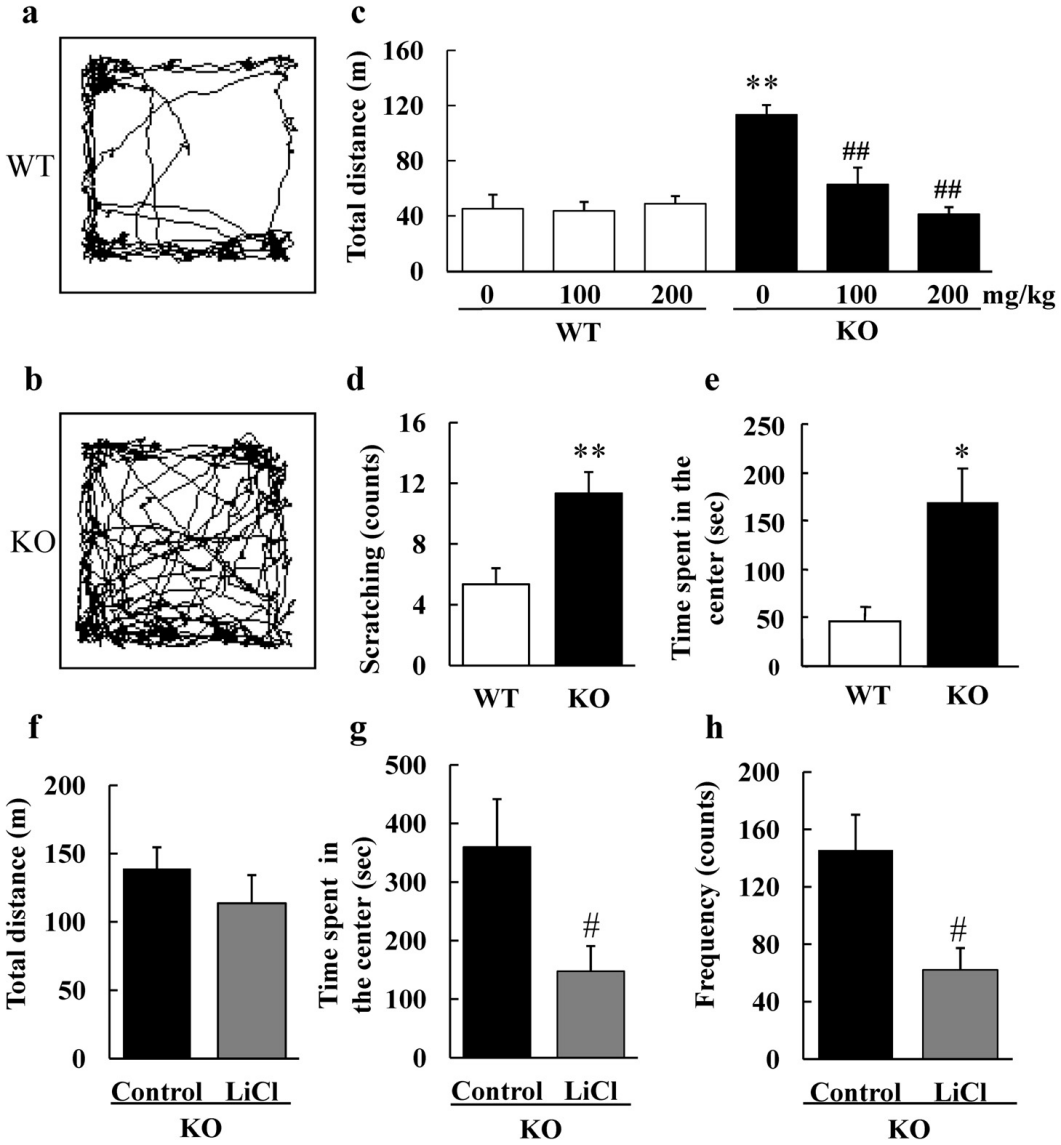
Open field test of WT and DGKβ KO mice. (a–e) Assessment of the single-dose lithium effect on DGKβ KO mice in the open field test. Representative images show typical examples of WT (a) and DGKβ KO (b) mice exploring behavior in the open field test. After drug treatment, each group of mice was placed in the open field apparatus and their distance traveled (c), number of scratching behaviors (d) and time spent in the center area (e) were measured. (n = 5 to 9) ^*^; p<0.05, ^**^; p<0.01 vs. WT mice. (f–h) Assessment of the chronic lithium effect on DGKβ KO mice in the open field test. After 10-days of drug treatment, mice were subjected to the open field test, and distance traveled (f), time spent in the center area (g) and the frequency of entering the center area (h) were measured for one hour. (n = 6 or 7) ^#^; p<0.05 vs. control mice.

Next, DGKβ KO mice were subjected to an open field test, and their activity and stereotyped behavior in the novel environment was compared. Total travel distance and the number of scratching behaviors were significantly greater in KO mice than in WT mice (Figures 2a–d). Moreover, administration of a mood stabilizer, lithium revealed that the horizontal activity of lithium-treated DGKβ KO mice was decreased (Figure 2c). In comparison, this concentration of lithium did not affect WT mice locomotor (Figure 2c). On the other hand, other drugs, such as haloperidol (0.1 mg/kg, i.p.), diazepam (1.0 mg/kg, i.p.), or imipramine (20 mg/kg, i.p.), did not affect the lcomotoer activity of DGKβ KO mice (Figure S1a and d).

### DGKβ KO mice exhibited less anxiety

In open field test, we also assessed the anxiety level of DGKβ KO mice by measuring their stay time in the center area of open field apparatus (anxiety-provoking area for mice). In this assessment, DGKβ KO mice spent more time in the center area of the apparatus than WT mice (Figure 2e). To investigate the effect of lithium on fearless behavior of DGKβ KO mice, mice were given a low-dose of lithium for 10 days, and their anxiety level in an open field test was measured. Low-dose lithium treatment did not reduce the activity of DGKβ KO mice (Figure 2f). This paradigm of LiCl treatment produces a stable serum Li^+^ concentration [14] which is at the low end of the therapeutic range for human patients [15]. On the other hand, fearless behavior of lithium-treated DGKβ KO mice was significantly decreased when compared with vehicle-treated DGKβ KO mice (Figures 2g and h). No significant changes were observed in total distance moved, duration in center zone, or frequency to center zone between vehicle- and LiCl-treated WT mice (Figure S2a–c), which was consistent with previous reports [14].

**Figure 3:**
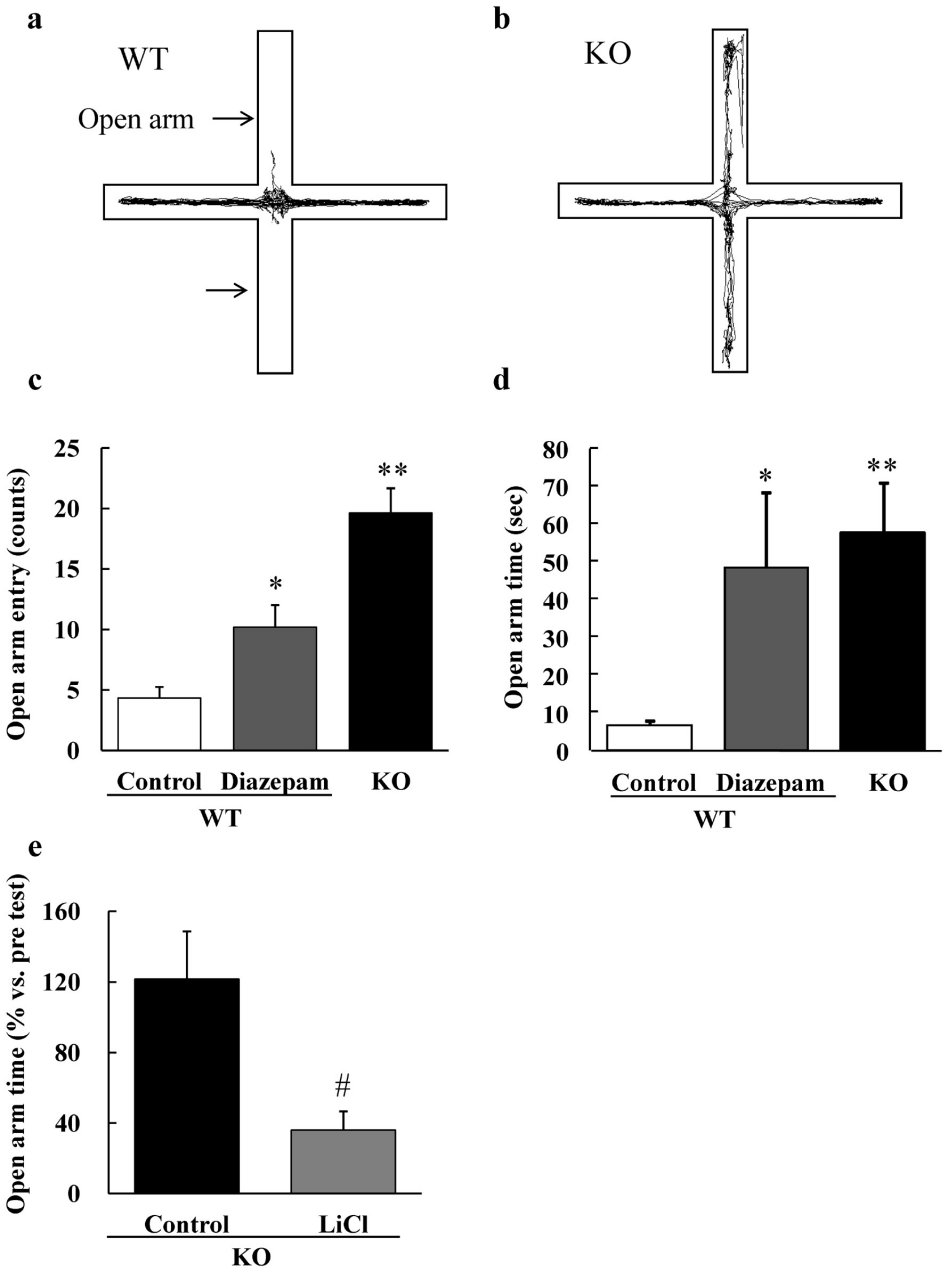
Elevated plus maze test of WT and DGKβ KO mice. Representative images show typical examples of WT (a) and DGKβ KO (b) mice exploring in the elevated plus maze apparatus. After drug treatment, each group of mice was placed in the elevated plus maze apparatus for 10 min, and the number of entries into (c) and the time spent in open arms (d) were assessed. (e) After 10-days of drug treatment, mice were subjected to the elevated plus maze test again, and their time spent in open arms as a ratio to the pre test was assessed. (n = 7 to 9) ^*^; p<0.05, ^**^; p<0.01 vs. WT mice. ^#^; p<0.05 vs. control mice.

To further assess anxiety levels of DGKβ KO mice, we conducted another anxiety measuring behavioral test, an elevated plus maze test. In this test, total distance moved on the apparatus was not different between genotypes (WT vs. KO: 13.2±1.1 vs. 13.7±2.0 m, n = 7). On the other hand, the number of entries into and the time spent in open arms of DGKβ KO mice were greater than vehicle-treated WT group, and at the same or larger levels than anti-anxiety drug-treated WT mice (Figures 3a–d). These results indicate that DGKβ KO mice showed less anxiety. In this test, low-dose lithium treatment also attenuated the time spent in open arms of DGKβ KO mice (Figure 3e). No significant change was also observed in duration in open arm between vehicle- and LiCl-treated WT mice (Figure S2d). On the other hand, haloperidol (0.1 mg/kg, i.p.), diazepam (1.0 mg/kg, i.p.), or imipramine (20 mg/kg, i.p.) did not affect the behavior of DGKβ KO mice in elevated plus maze test (Figure S1b, c, e, and f).

### DGKβ KO mice decreased immobility in forced swim test and tail suspension test

**Figure 4:**
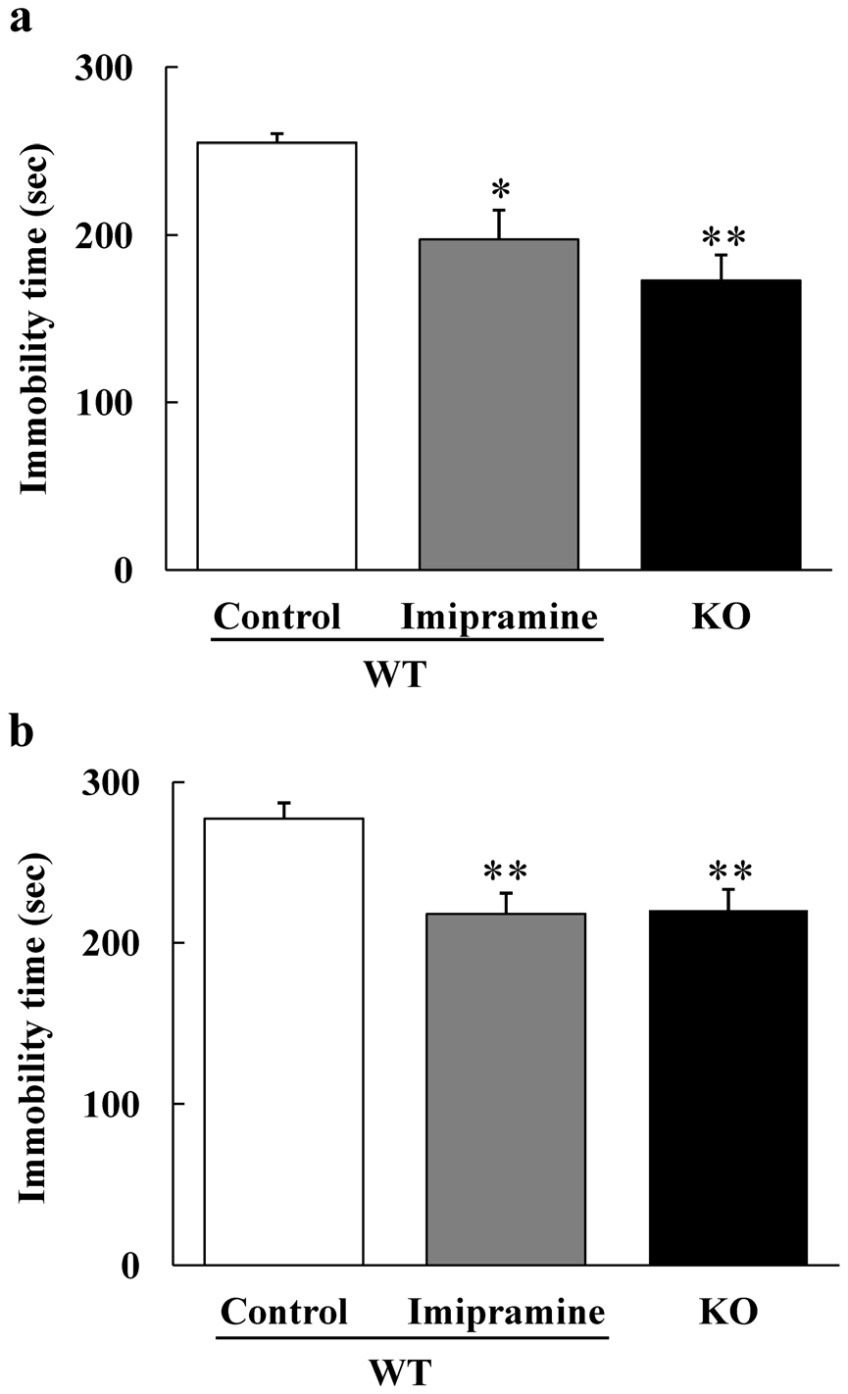
Antidepressant-like behaviors of DGKβ KO mice. (a) Immobile time of forced swim test. Thirty-minutes after drug treatment, mice were placed in water for a period of 6 min; only the last 5 min immobility time was measured. (b) Immobile time of tail suspension test. Thirty-minutes after drug treatment, mice were tail suspended with an adhesive tape 50 cm above the floor over a period of 7 min; immobility time was measured in only the last 6 min. (n = 7 to 9) ^*^; p<0.05, ^**^; p<0.01 vs. WT mice.

The mouse model for mood disorders generally exhibits changes in their depressive states, i.e. model mouse of mania exhibits lower depressive states [14]; we evaluated these changes in DGKβ KO mice using forced swim test and tail suspension test. In each test, both anti-depressant (imipramine)-treated WT mice and DGKβ KO mice showed a reduction in the despair state, as assessed by immobility time in these tests (Figures 4a and b).

### DGKβ KO mice did not exhibit impairment in sensorimotor gating and social interaction

To further analyze the effects of DGKβ deficits on psychomotor behavior, we evaluated schizophrenic-like behaviors in DGKβ KO mice using a prepulse inhibition (PPI) test and a social interaction test (Method S1). In the PPI test, sensorimotor gating can be assessed following PPI of the startle reflex, which is the modulation of the startle response, following a weak prepulse. Sensorimotor gating is the neural process which allows attention to be focused on one stimulus. DGKβ KO mice showed normal responses to both startle amplitude for the pulse-only trial (Figure S3a) and PPI (Figure S3b). In a social interaction test, we assessed social affiliative behavior in DGKβ KO mice, and the results revealed that DGKβ KO mice also exhibited normal social interaction (Figure S3c).

### Phosphorylation levels of Akt and GSK3β

**Figure 5:**
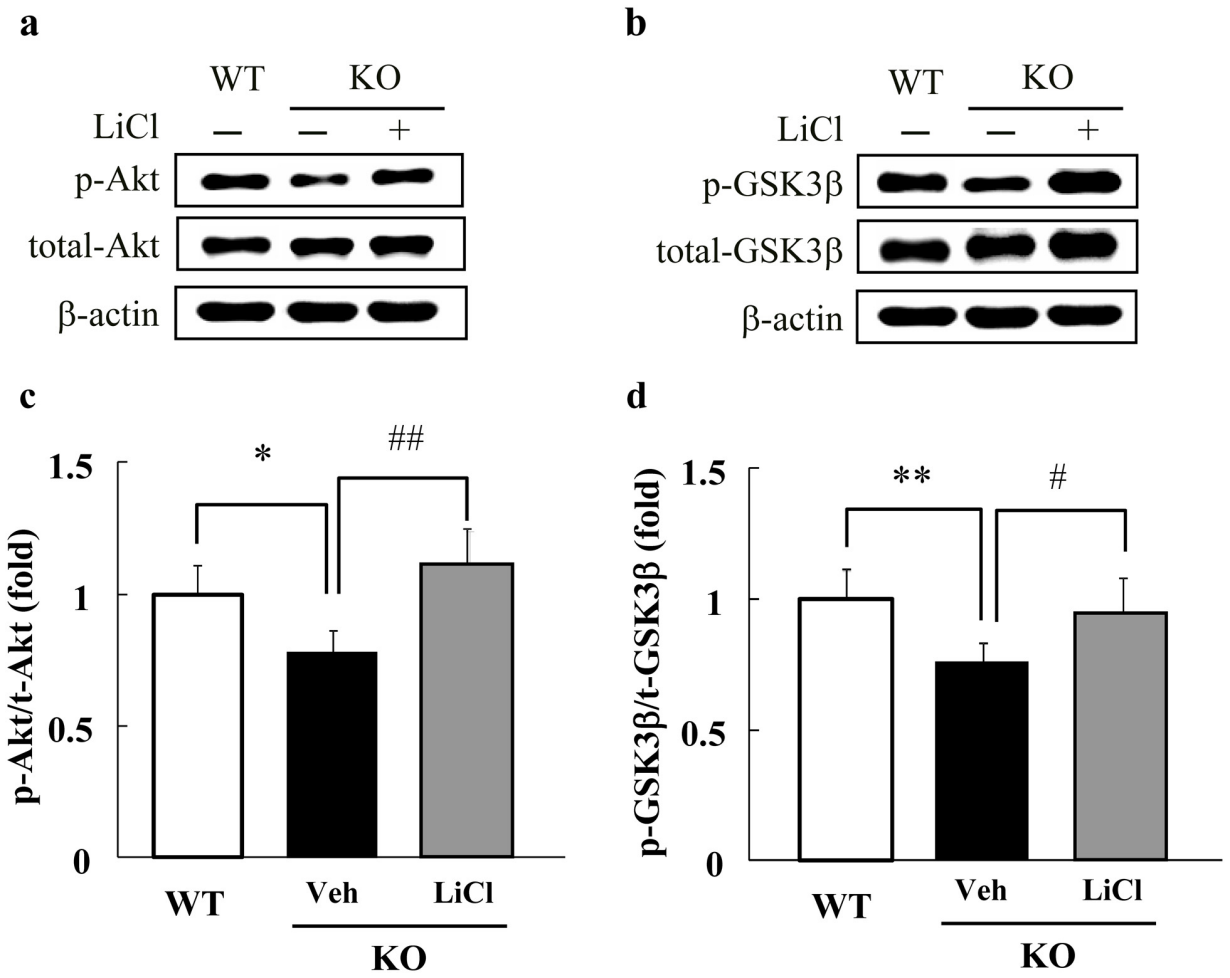
Western blot analysis of Akt-GSK3β signaling. (a, b) Phosphorylated Akt (Ser473) and GSK3β were decreased in the cortex of control group of DGKβ KO mice. (c, d) Quantitative analysis of Western blotting showed that phosphorylated Akt (Ser473) and GSK3β were decreased in the cortex of DGKβ KO mice, and that lithium treatment attenuated this effect. (n = 7) ^*^; p<0.05, ^**^; p<0.01 vs. WT mice. ^#^; p<0.05, ^##^; p<0.01 vs. control mice.

Using Western blot analysis, we evaluated the phosohorylation levels of Akt and GSK3β, which are the downstream of the proteins PKC and PA. Moreover, these proteins were reported as one of the pharmacological action mechanisms of lithium [16], [17]. The total and phosohorylation levels of Akt and GSK3β proteins in the hippocampus and striatum were not different between WT mice and DGKβ KO mice (data not shown). However, the phosphorylation levels of Akt (Ser473) and GSK3β were decreased in the cortex of DGKβ KO mice despite the normal levels of total Akt and GSK3β proteins (Figures 5a–d). Furthermore, the treatment of lithium attenuated these reductions in the phosphorylated proteins (Figures 5a–d). The phosphorylation level of Akt (Thr308) was also decreased in the cortex of DGKβ KO mice, compared with WT mice [WT; 1.00±0.13 (fold) (mean ± S.E.M., n = 7), KO; 0.25±0.04 ^**^ (n = 7), ^**^; p<0.01 vs. WT]. No significant change was observed in the phosphorylation level of Akt (Ser473) or GSK3α/β between vehicle- and LiCl-treated WT mice (Figure S4a–d). We have additionally investigated the effect of haloperidol on the phosphorylation levels of Akt or GSK3α/β in the cortex. In these results, haloperidol (0.1 mg/kg, i.p.) did not alter the decreased phosphorylation levels of Akt (Ser473), GSK3α, or GSK3β in DGKβ KO mice (Figure S5a–e).

### Morphological changes in spine formation of the cortical neuron

**Figure 6:**
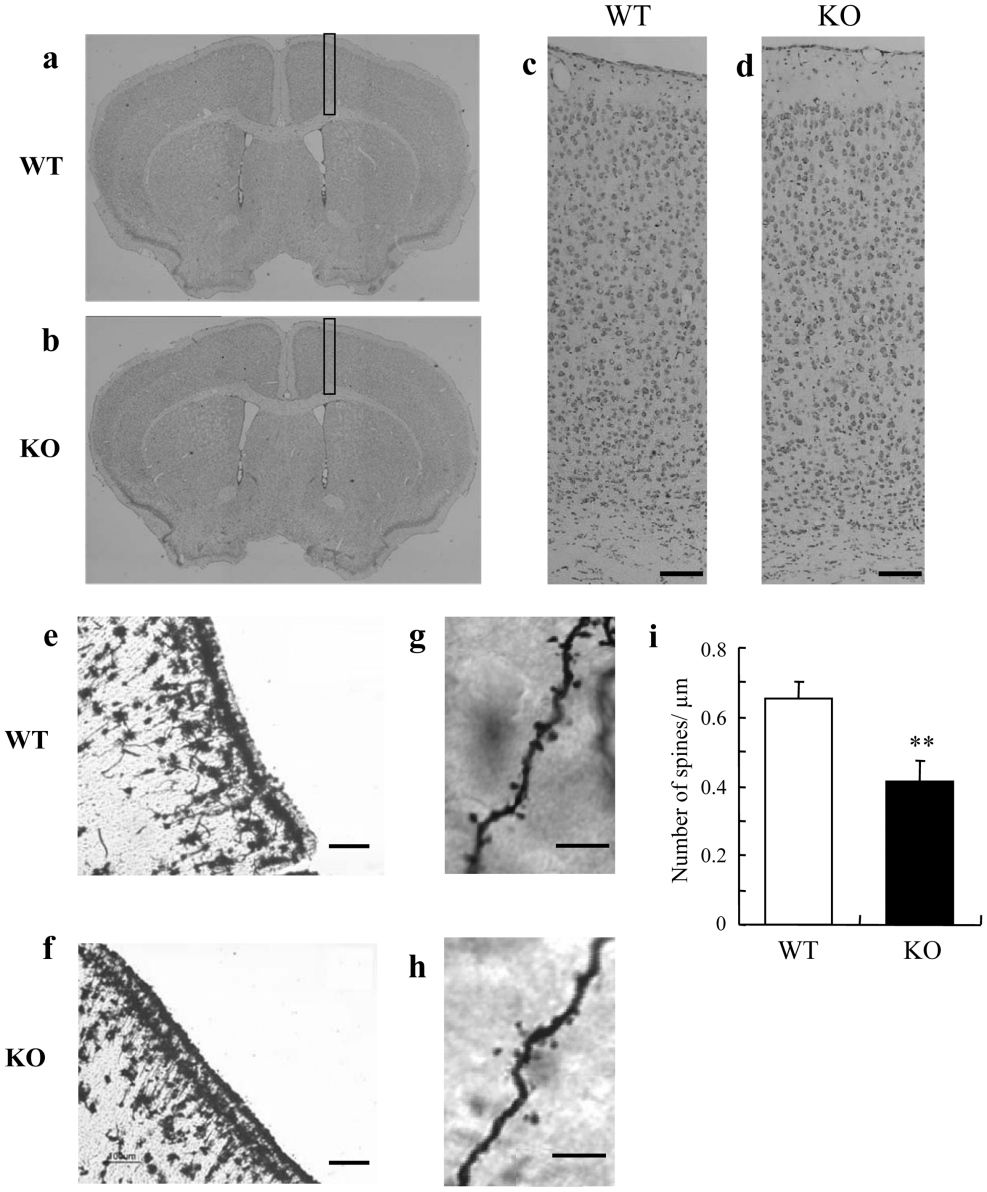
Histological analysis of the cortex in DGKβ KO mice. (a–d) Cresyl violet staining. (a, b) Representative photomicrographs show coronal sections stained with cresyl violet. (c, d) DGKβ KO mice showed no defects in the layered structure of the cerebral cortex. Scale bar  = 100 µm (e–i) Golgi staining. (e, f) Representative photomicrographs show pyramidal neurons in the cortex stained by Golgi. Scale bar  = 100 µm (g, h) Representative photomicrographs show high-magnification images of apical dendritic segments. Scale bar  = 5 µm (i) Quantitative analysis of spine density in WT mice and DGKβ KO mice. (n = 11 or 15) ^**^; p<0.01 vs.WT mice.

To investigate the morphological factors relating to behavioral changes and the decrease in the phoshorylation levels of Akt and GSK3β in cortex, we first analyzed brain sections stained with cresyl violet. In this analysis, DGKβ KO mice showed normal cortical laminar structure (Figures 6a–d). On the other hand, the results of Golgi staining revealed that the cortical spine density of DGKβ KO mice was significantly decreased in comparison with that of WT mice (Figures 6 e–i).

## Discussion

In the present study, we performed a comprehensive behavioral analysis of DGKβ KO mice in order to investigate the role of DGKβ in higher brain functions and the relationship between DGKβ and bipolar disorder.

In the locomotor activity and open field tests, DGKβ KO mice exhibited hyperactivity in their home cage and in a novel environment. Increased locomotor activities were observed in other rodent models of mental disease, such as during the mania state of bipolar disorder [18], attention deficit hyperactive disorder (ADHD) [16], and positive symptoms of schizophrenia [19], [20]. In the open field test, LiCl at 100 mg/kg or more significantly attenuated the hyperactivity of DGKβ KO mice. It is known that LiCl at these doses does not cause toxicity to mice [21] and, in the present study, LiCl did not affect WT mice locomotor behavior. On the other hand, haloperidol, diazepam, or imipramine did not improve locomotor behaviors in DGKβ KO mice. These results indicate that the hyperactivity of DGKβ KO mice is a lithium-sensitive behavioral abnormality, similar to the mania exhibited in some animal models. DGKβ KO mice also displayed more frequent scratching behavior than WT mice. Stereotypical behaviors such as scratching and head twitching are induced by the administration of psychostimulants [22], and it is also observed in mouse model of psychiatric disorders [23].

Using an open field test or an elevated plus maze test, one is able to evaluate anxiety levels in rodent models. In the open field test, DGKβ KO mice spent more time in the center of the apparatus than WT mice, indicating that DGKβ KO mice showed less-anxiety. Moreover, chronic treatment with a low-dose (600 mg/L) of lithium decreased fearless behavior in KO mice without decreasing their locomotor activity. With this dose of LiCl treatment, a serum concentration of Li^+^ has reported to be stable at approximately 0.41 mmol/L [14], a concentration which is typically used to treat mania patients [15]. When the effects of the drug on animal anxiety level are measured, a low concentration is recommended because high concentration of LiCl causes a decrease in mice locomotor activity. In an elevated plus maze test, DGKβ KO mice displayed enhanced open arm selectivity, which also indicates lowered anxiety of DGKβ KO mice. Chronic LiCl treatments in DGKβ KO mice also attenuated these behavioral changes. During each session, the activity of each group of mice was not different significantly, and therefore these results verify that lithium specifically inhibits fearless behaviors of DGKβ KO mice in a manner not due to change in their locomotor activity. These lithium-sensitive changes of anxiety in mice are observed especially in the animal model of mania [14].

Forced swim test and tail suspension test are commonly used to evaluate depression-like behaviors of rodent models. Because existing antidepressants specifically inhibit the immobile time in these tests, the utility of these experiments is recognized. In the present study, DGKβ KO mice exhibited antidepressant-like behavioral changes in both tests.

In addition to the variety of behavioral changes shown above, we previously investigated DGKβ KO mice exhibiting impaired memory [8]. The cognitive functioning of DGKβ KO mice were analyzed, and these were less affected by locomotor activity or swimming ability [8]. Therefore, the relationship between hyperactivity and memory impairment in DGKβ KO mice is weak. On the other hand, DGKβ KO mice did not show schizophrenia-like behavioral changes, as typified by PPI and social interaction deficits (Figure S3). Taken together, DGKβ KO mice present lithium-sensitive excitatory psychomotor effects related to their mood state and cognitive impairment. These characteristics of behavior are similar to those observed in mouse models of mania [14] and ADHD [16].

Next, to analyze the mechanisms of abnormal behavior generation in DGKβ KO mice and the lithium sensitive mechanisms in DGKβ KO mice, we assessed the phosophorylation levels of Akt and GSK3β, which lie downstream of PKC and PA. The activity of DGK to catalyze the phosphorylation of DG into PA is an intrinsic component of the phosphatidylinositol cycle. Additionally, PA contributes to the activation of Akt/phosphatidylinositol 3-kinase (PI3K) signaling by stimulating PI4P5-kinase [24]. Indeed, using DGKζ-deficient mice, DGKζ has been reported to be involved in the PI3K/Akt pathway [25] and phosphorylated Akt inhibits the activity of GSK3β by phosphorylating at Ser9 [26], [27]. It was reported that Akt and GSK3β are one of target proteins of lithium; lithium increases the phosphorylation levels of Akt and GSK3β [16], [28], [29], [30], [31]. In the present study, the phosophorylation levels of Akt (Ser473) and GSK3β were decreased in the cortex of DGKβ KO mice. In addition, lithium significantly attenuated the alteration of these phosophorylation levels in DGKβ KO mice. The phosphorylation level of Akt (Thr308) was also decreased in the cortex of DGKβ KO mice, compared with WT mice. These decreases in levels of phosphorylated Akt and GSK3β were observed in the striatum of dopamine transporter (DAT) KO mice [16]. The effects of lithium on phosphorylated Akt and GSK3β in DGKβ KO mice are also similar to those observed in DAT KO mice [16]. Transgenic mice overexpressing GSK3β also display mania-like behaviors [32]. Additionally, β-catenin, the downstream protein of GSK3β over expression mice exhibit lithium-sensitive behaviors [33]. On the other hand, lithium attenuates the behavior of GSK3β heterogeneous mice in locomoter activity and tail suspension test [34]. We have also investigated the changes of phospho-GSK3α and tau hyperphosphorylation (using the AT8 antibody) in the cortex of WT and DGKβ KO mice. The protein level phospho-GSK3α was decreased in DGKβ KO mice compared with WT mice (Figure S5c). On the other hand, the level of tau hyperphosphorylation in DGKβ KO mice trended to increase, but did not reach a significant level (Figure S4e). Although we can not fully explain the reason that the level of tau hyperphosphorylation was not significant, it is possible that the phosphorylation of tau may be regulated by multiple kinases. Especially, tau pshosphorylation at Ser 202 (detected by AT8) is regulated not only by GSK3β, but also by cyclin-dependent protein kinase 5 (cdk5) [35]. On the other hand, GSK activity reflected the ratio of phosphor-Ser9 GSK3β to total GSK3β levels, as others reported [36]. These results suggest that the alteration of GSK3β function affected mood-behavior in mice, and the lithium-sensitive excitatory mood behaviors of DGKβ KO mice are partly caused by the reduced phosphorylation levels of Akt and GSK3β. Some reports have shown that lithium affects both Akt and GSK3 phosphorylation in the striatum of WT animals [36]. On the other hand, in the present study we focused solely on the cortical changes of Akt and GSK3 phosphorylation. Effects of lithium on Akt and GSK3 phosphorylation may vary depending upon the brain region or the mouse strain. Additionally, our results showing that LiCl (200 mg/kg i.p.) did not affect locomotor activity in WT mice (Figure 2c) may support the results of Akt and GSK3 phosphorylation in WT cortex.

Previously, we and other groups have reported the contribution of DGKβ to hippocampal spine formation [7], [8]. Our present study revealed that impaired spine formation also occurred in the cortex of DGKβ KO mice without changing cortical laminar structure. The decrease of spine density in cortex was observed in many animal models expressing abnormal psychomotor behaviors [20], [37], [38]. In addition, postmortem brain analysis revealed that spine density on the primary apical dendrites of the cortex layer III pyramidal neurons were also decreased in psychiatric diseased patients [39], [40]. As the reports implied, dendrite spine dysplasia led to the expression of abnormal behavior, and in DGKβ KO mice the decrease of cortical spine density, in turn, may have led to the observed abnormalities in behavior.

In conclusion, DGKβ KO mice exhibited lithium-sensitive excitatory psychomotor behaviors related to their mood state. The phenotypes of DGKβ KO mice may be caused, at least in part, by the impairment of Akt-GSK3β signaling and cortical spine dysplasia.

## Supporting Information

Method S1 Prepulse inhibition and social interaction test.
(0.03 MB DOC)

Figure S1 Effects of haloperidol, imipramine, and diazepam in open field test and elevated plus maze test. Haloperidol (0.1 mg/kg, i.p.), diazepam (1.0 mg/kg, i.p.), imipramine (20 mg/kg, i.p.), or vehicle was administrated at 30 min before the behavioral trial. (a–c) The effects of haloperidol (0.1 mg/kg, i.p.) in open field test and elevated plus maze test. (a) Distance moved was measured in open field test (n = 8 to10). (b) Time spent in and (c) frequency to open arms were measured in elevated plus maze test (n = 7 to 9). Veh; vehicle, Hal; haloperidol, *; p<0.05 vs. vehicle-treated WT mice. (d–f) The effects of diazepam (1.0 mg/kg, i.p.) and imipramine (20 mg/kg, i.p.) in open field test and elevated plus maze test. (d) Distance moved was measured in open field test (n = 6 or 7). (e) Time spent in and (f) frequency to open arms were measured in elevated plus maze test (n = 5 to 12). Imi; imipramine, Dia; diazepam, *; p<0.05, **; p<0.01 vs. vehicle-treated WT mice.
(4.21 MB TIF)

Figure S2 Effects of chronic LiCl administration on the behavior of WT mice. For assessment of chronic lithium treatment, LiCl was mixed into the drinking water at 600 mg/L and given for 10 days. Controls were given normal water. After the 10-days drug treatment, WT mice were subjected to the open field test and elevated plus maze test. (a) Distance moved, (b) duration in, and (c) frequency into center zone were measured in open field test (n = 12 and 13). (d) Time spent in open arms, as a ratio to the pre test, in elevated plus maze test (n = 11).
(3.84 MB TIF)

Figure S3 Prepulse inhibition and social interaction test in DGKβ KO mice. (a, b) PPI of acoustic startle response in WT (n = 7) and KO mice (n = 9). (a) In the 120 dB-pulse-only trials, startle amplitude did not differ significantly between DGKβ KO and WT mice. (b) The PPI is expressed as a percentage of the startle response to a 120 dB-pulse. DGKβ KO mice showed normal PPI at each prepulse intensity. (c) Social interaction test in a novel environment in WT (n = 8) and KO (n = 8) mice. Two genetically identical mice that had been housed separately were placed in the same cage. Their social interaction was then monitored for 10 min. There was no significant difference in duration per contact between WT and DGKβ KO mice.
(1.03 MB TIF)

Figure S4 Western blot analysis in Akt-GSK3β signaling. Effects of LiCl on Akt-GSK3β signaling in the cortex of WT mice were measured (a–e). LiCl (200 mg/kg, i.p.) or vehicle was administrated at 30 min before the western blotting. (a) Representative images of immunoblottin showing p-GSK3α/β, total GSK3α/β, p-Akt (Ser473), total Akt, and β-actin. Quantitative analysis of (b) p-GSK3α/GSK3α, (c) p-GSK3β/GSK3β, and (d) p-Akt (Ser473)/Akt (n = 7). (e) Tau phosphorylation (using the AT8 antibody) in the cortex of WT and DGKβ KO mice (n = 5 and 6).
(3.61 MB TIF)

Figure S5 Effects of haloperidol on Akt-GSK3β signaling in the cortex of WT and DGKβ KO mice. Haloperidol (0.1 mg/kg, i.p.) or vehicle was administrated at 30 min before the western blotting. Representative images of immunoblottin showing (a) p-GSK3α/β and total GSK3α/β, and (b) p-Akt (Ser473) and total Akt. Quantitative analysis of (c) p-GSK3α/GSK3α, (d) p-GSK3β/GSK3β, and (e) p-Akt (Ser473)/Akt. (n = 5 and 8). Veh; vehicle, Hal; haloperidol, *; p<0.05, **; p<0.01 vs. vehicle-treated WT mice.
(4.03 MB TIF)
