# Supplementary material for: Diacylglycerol Kinase β Knockout Mice Exhibit Lithium-Sensitive Behavioral Abnormalities
Source: PLoS One. 2010 Oct 18;5(10):e13447. doi: 10.1371/journal.pone.0013447 (PMC2956634; doi:10.1371/journal.pone.0013447)
Supplement: Method S1 — Prepulse inhibition and social interaction test. (0.03 MB DOC) [file pone.0013447.s001.doc]

**Supporting information methods**

**PPI test**

Acoustic startle responses were measured in a startle chamber (SR-LAB; San Diego Instruments, San Diego, CA, USA). Mice were presented with a series of four discrete trials: pulse-alone trials, prepulse-plus-pulse trials, prepulse-alone trials, and trials in which no discrete stimulus, other than the constant background noise, was presented. A reduction in startle magnitude in the prepulse-plus-pulse trials relative to that in the pulse-alone trials constitutes PPI. The pulse stimulus employed was 120 dB in intensity and 40 msec in duration. Prepulses of various intensities were employed: 73, 76, and 82 dB. The duration of the prepulse stimuli was 20 msec. The stimulus onset asynchrony of the prepulse and pulse stimuli in the prepulse-plus-pulse trial was 100 msec. A session began with the animals being placed into a Plexiglass enclosure. They were acclimatized to the apparatus for 5 min before the first trial began. The mice were presented with 3 blocks of discrete test trials. Each block consisted of one trial of each of the following trial types: pulse-alone trials, prepulse-plus-pulse trials with each of the three levels of prepulse, prepulse-alone trials with each of the three levels of prepulse, and no stimulus trials (i.e., background noise alone). The interval between successive trials was variable, the mean being 30 sec (range, 20 to 40 sec).

**Social interaction test**

Two mice of identical genotypes that had previously been housed in different cages were placed in a box together (length 17.5 × width 24.5 × height 12.5 cm) and allowed to explore freely for 10 min. Social behavior was monitored by means of a video camera (Everio; Victor, Yokohama, Japan). The number and mean duration of contacts were measured at 10 min after the start.
