# Supplementary figures and images for: Diacylglycerol Kinase β Knockout Mice Exhibit Lithium-Sensitive Behavioral Abnormalities
Source: PLoS One. 2010 Oct 18;5(10):e13447. doi: 10.1371/journal.pone.0013447 (PMC2956634; doi:10.1371/journal.pone.0013447)

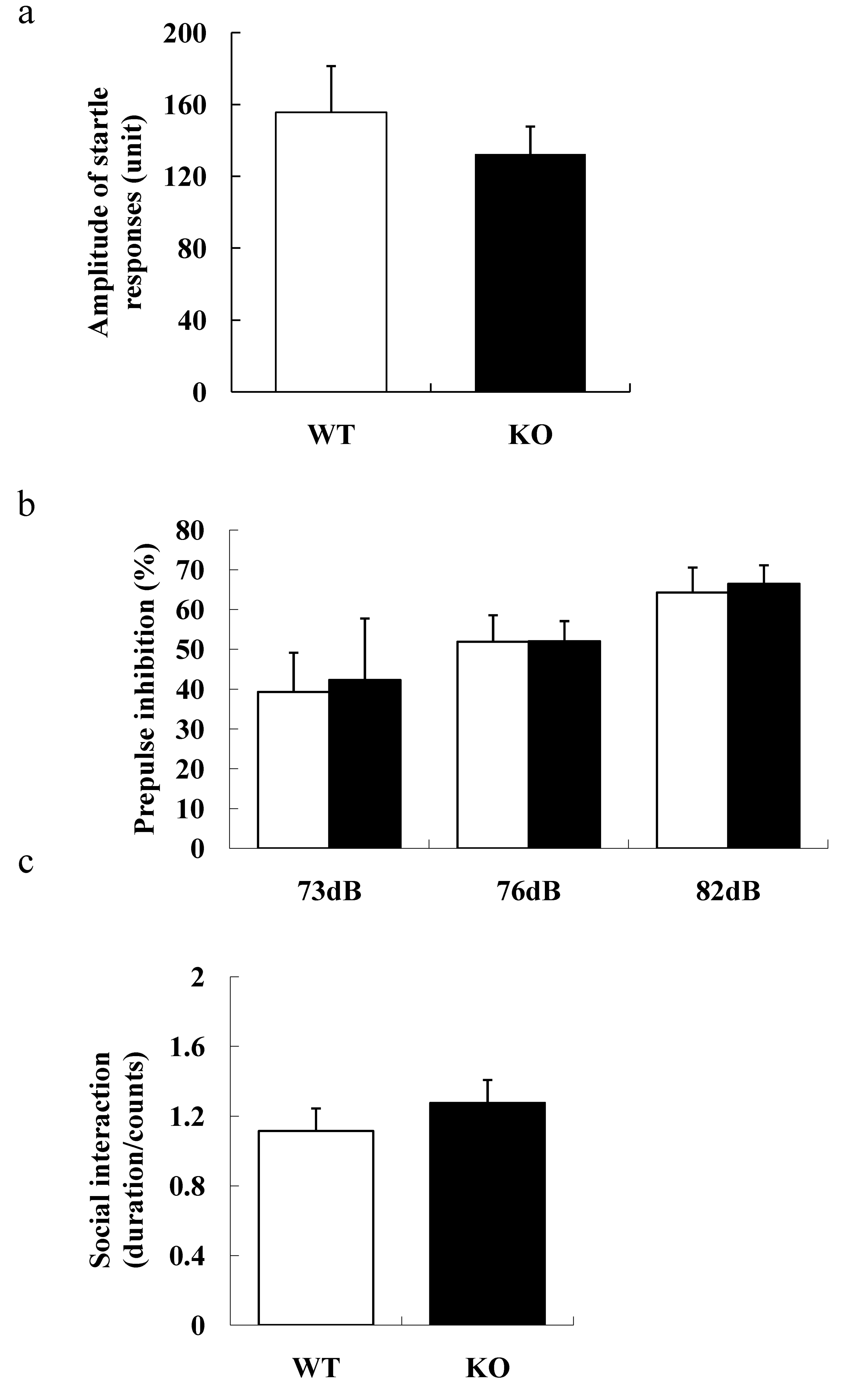

Supplement: Figure S3 — Prepulse inhibition and social interaction test in DGKβ KO mice. (a, b) PPI of acoustic startle response in WT (n = 7) and KO mice (n = 9). (a) In the 120 dB-pulse-only trials, startle amplitude did not differ significantly between DGKβ KO and WT mice. (b) The PPI is expressed as a percentage of the startle response to a 120 dB-pulse. DGKβ KO mice showed normal PPI at each prepulse intensity. (c) Social interaction test in a novel environment in WT (n = 8) and KO (n = 8) mice. Two genetically identical mice that had been housed separately were placed in the same cage. Their social interaction was then monitored for 10 min. There was no significant difference in duration per contact between WT and DGKβ KO mice. (1.03 MB TIF) [file pone.0013447.s004.tif]

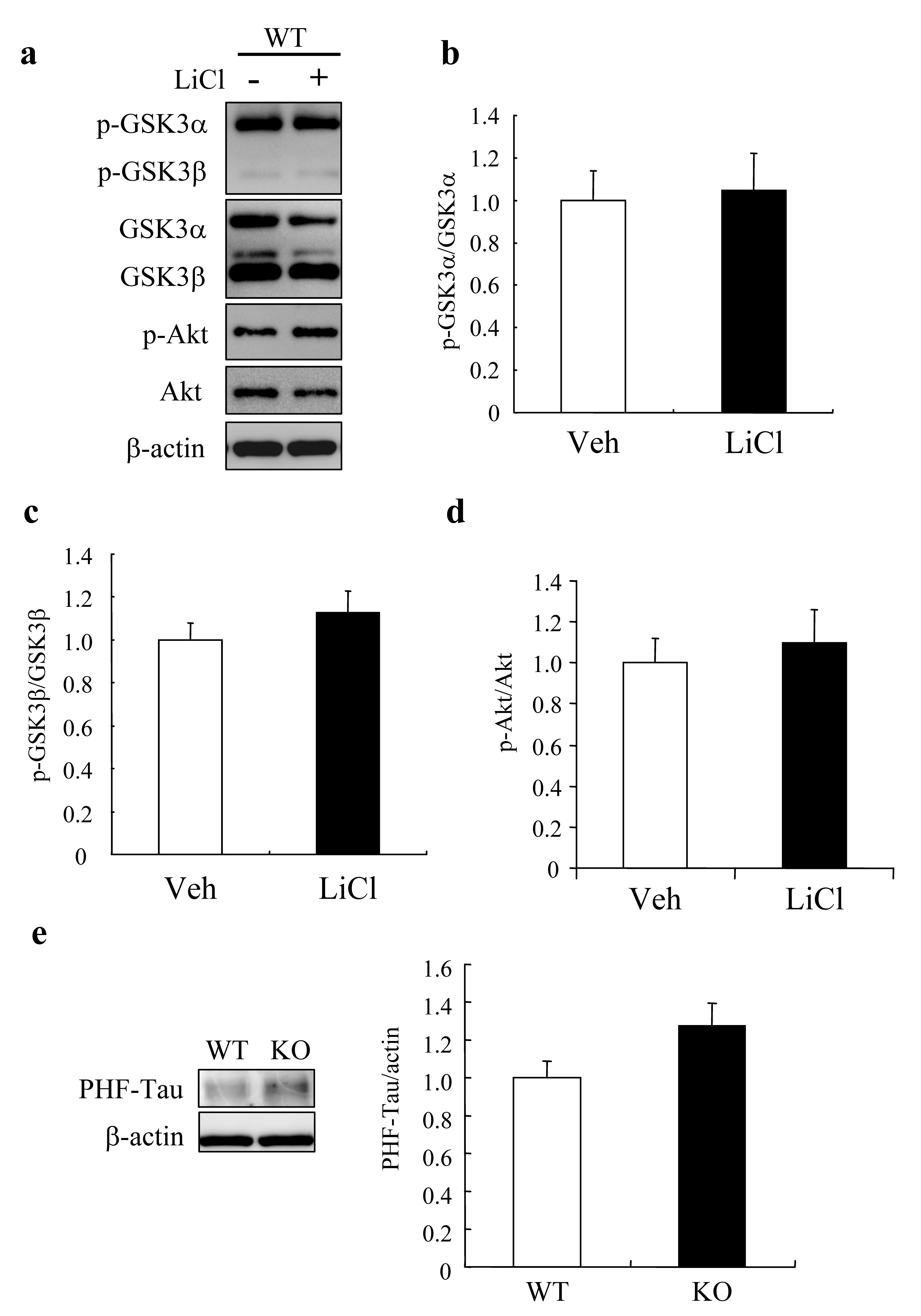

Supplement: Figure S4 — Western blot analysis in Akt-GSK3β signaling. Effects of LiCl on Akt-GSK3β signaling in the cortex of WT mice were measured (a–e). LiCl (200 mg/kg, i.p.) or vehicle was administrated at 30 min before the western blotting. (a) Representative images of immunoblottin showing p-GSK3α/β, total GSK3α/β, p-Akt (Ser473), total Akt, and β-actin. Quantitative analysis of (b) p-GSK3α/GSK3α, (c) p-GSK3β/GSK3β, and (d) p-Akt (Ser473)/Akt (n = 7). (e) Tau phosphorylation (using the AT8 antibody) in the cortex of WT and DGKβ KO mice (n = 5 and 6). (3.61 MB TIF) [file pone.0013447.s005.tif]

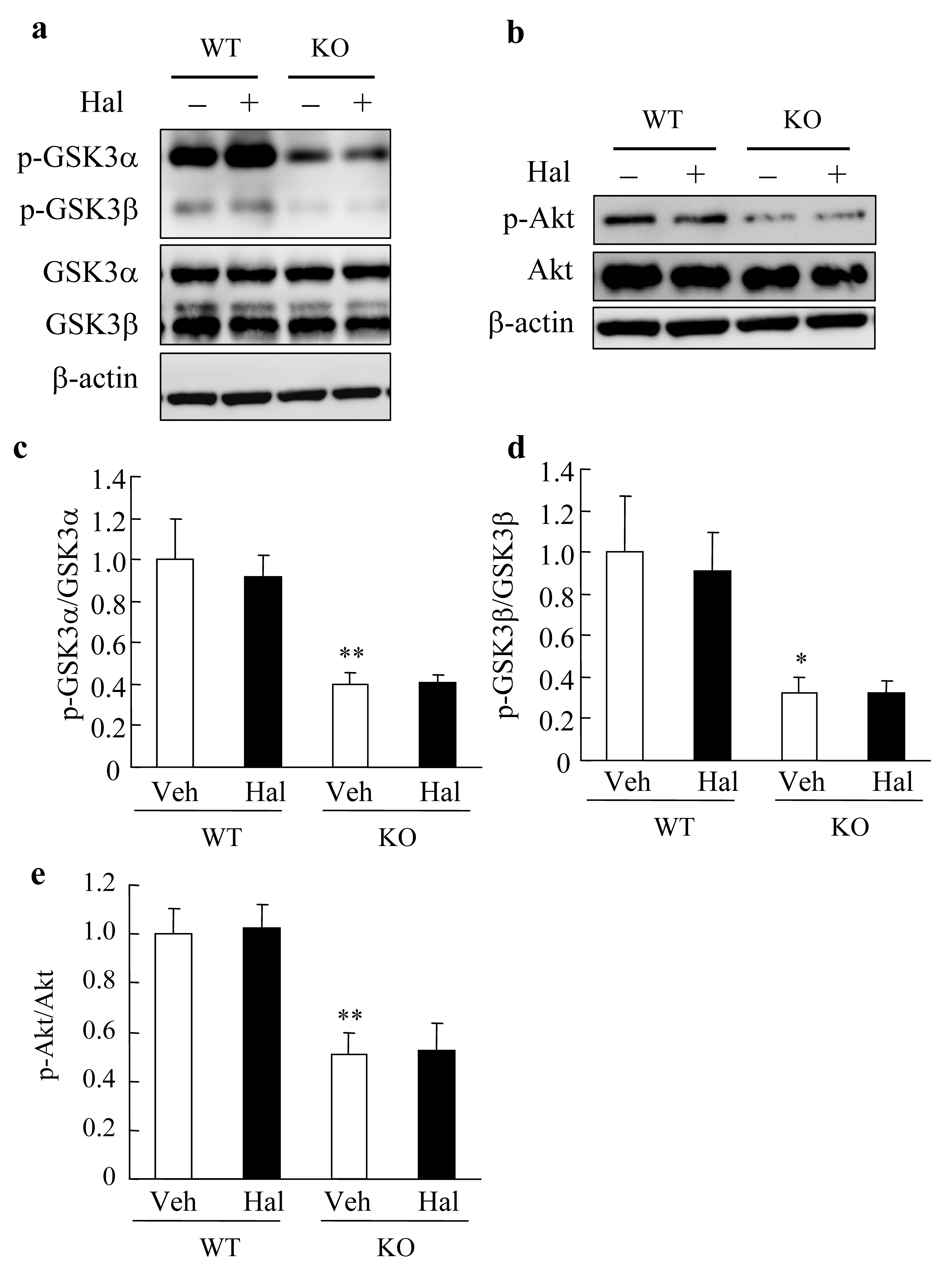

Supplement: Figure S5 — Effects of haloperidol on Akt-GSK3β signaling in the cortex of WT and DGKβ KO mice. Haloperidol (0.1 mg/kg, i.p.) or vehicle was administrated at 30 min before the western blotting. Representative images of immunoblottin showing (a) p-GSK3α/β and total GSK3α/β, and (b) p-Akt (Ser473) and total Akt. Quantitative analysis of (c) p-GSK3α/GSK3α, (d) p-GSK3β/GSK3β, and (e) p-Akt (Ser473)/Akt. (n = 5 and 8). Veh; vehicle, Hal; haloperidol, *; p<0.05, **; p<0.01 vs. vehicle-treated WT mice. (4.03 MB TIF) [file pone.0013447.s006.tif]
